# Methods for detection and identification of beer-spoilage microbes

**DOI:** 10.3389/fmicb.2023.1217704

**Published:** 2023-08-10

**Authors:** Ryanne C. Oldham, Michael A. Held

**Affiliations:** ^1^Department of Chemistry and Biochemistry, Ohio University, Athens, OH, United States; ^2^Quality Assurance and Quality Control Laboratory, Jackie O’s Brewery, Athens, OH, United States; ^3^Molecular and Cellular Biology Program, Ohio University, Athens, OH, United States

**Keywords:** BSM, VBNC, LAB, qPCR, primer, DNA extraction

## Abstract

It is critical that breweries of all sizes routinely monitor the microbiome of their process to limit financial losses due to microbial contamination. Contamination by beer-spoiling microbes (BSMs) at any point during the brewing process may lead to significant losses for breweries if gone undetected and allowed to spread. Testing and detection of BSMs must be routine and rapid, and because even small breweries need the capability of BSM detection and identification, the method also needs to be affordable. Lactic acid bacteria (LAB) are responsible for most spoilage incidents, many of which have been shown to enter the viable but nonculturable (VBNC) state under conditions present in beer such as cold or oxidative stress. These bacteria are invisible to traditional methods of detection using selective media. This article describes several methods of BSM detection and identification that may be useful in the majority of craft breweries. While there are several genomic methods that meet some or many qualifications of being useful in craft breweries, real-time quantitative polymerase chain reaction (qPCR) currently best meets the desired method characteristics and holds the most utility in this industry, specifically SYBR Green qPCR. qPCR is a targeted method of detection and identification of microbes that is affordable, rapid, specific, sensitive, quantitative, and reliable, and when paired with valid DNA extraction techniques can be used to detect BSMs, including those in the VBNC state.

## 1. Introduction

Due to the intrinsic antimicrobial properties of beer, the growth of pathogens and many other bacteria are inhibited. Growth-inhibiting characteristics of beer include low pH, the presence of ethanol, carbon dioxide, antibacterial compounds from hops (e.g., iso-α acids), and low nutrient and oxygen availability (Sakamoto and Konings, 2003; Priest and Campbell, 2009; Bokulich et al., 2015; Hill, 2015). However still, microbial contamination in the brewery is a major concern, as it can lead to product spoilage and significant profit loss. Various microorganisms that may be introduced to beer during the production and/or packaging process metabolize beer compounds, producing off-flavors which remain present in the final product and diminish overall beer quality and stability. Thus, it is necessary for beer manufacturers to routinely monitor the microflora of surfaces and liquid samples throughout the beer-making process (Hill, 2015; Pellettieri, 2015).

Additionally, contamination may spread quickly throughout a brewery if undetected for even a short period of time; yeast is either propagated or harvested from one tank and used in another, batches may be blended into a single tank, and different batches flow through the same equipment. Breweries must be capable of rapidly detecting and identifying BSMs throughout the entire process of producing, packaging, and selling beer. The risk of contamination spreading and ultimately product and financial losses to the brewery decrease the sooner a contamination is detected. Because it is essential to detect contamination as soon as possible, sampling and testing should be frequent and routine.

Methods of microbial detection/identification for successful and routine testing in most brewery settings should be (1) rapid, (2) specific, (3) reliable (accurate), (4) quantifiable, (5) sensitive, and (6) affordable. Such methods could have many applications in a wide range of industries besides brewing including medical, pharmaceutical, water treatment, biological, virology, epidemiology, forensics, food and beverage, agricultural, and other fermentation processes.

Barrel aged beers generally hold a higher profit value for breweries; unique flavor profiles, high alcohol content (ABV), along with the time and space put into creating these beers allow breweries to increase retail prices of these brands. Often, consumers prefer to age (or “cellar”) purchased bottles for longer periods of time, sometimes for years, to develop unique flavor profiles and for the experience of savoring the products for years to come (Gonzalez, 2014). This is generally untrue for other beer styles, such as India Pale Ale (IPA), which most consumers prefer to drink as fresh as possible. Extended aging by consumers of high-cost barrel aged products increases the likelihood that any microbial contamination present will be given the opportunity to grow and metabolize beer compounds into off-flavors, eventually spoiling the product. Many breweries utilize pasteurization to decrease microbial populations in these beers prior to packaging. However, many small-to-medium-sized craft breweries are not able to afford pasteurization equipment. Additionally, the process of pasteurization may alter flavor profiles. Likewise, it is important for producers to be aware that although pasteurization will decrease the microbial load, it is not sterilization, and some microbes may still survive the process (Hill, 2015).

Thus, it is imperative to be able to detect low levels of BSMs prior to product sale throughout all stages of the barrel aging process. Failure to detect contamination, often by viable but nonculturable (VBNC) microbes, prior to product sale has led to costly recalls, labor-intensive refund programs, large profit losses, and brand reputation damage for several breweries (Noel, 2016).

There are several characteristics that methods of detection and identification of BSMs should have to be most useful in a brewery. To be most practical, the method(s) of BSM detection and identification should be rapid, specific, reliable (accurate), quantitative, sensitive, and affordable. Even better, the method should ideally be relatively simple to use and require minimal training. Because ingredients and products are transferred frequently throughout the brewery which increases the risk of spreading contaminants, early detection is key to preventing the problem from spreading and growing. Additionally, products that may be aged for extended periods of time are especially vulnerable to problems from even low levels of contamination. Therefore, the brewery’s method of BSM detection/identification needs to be sensitive to low-levels of contamination that may grow over time and should deliver rapid results. Because frequent routine sampling and testing is key to early detection, it is something that must be done by even the smallest breweries to prevent financial losses due to microbial contamination. Many craft breweries are working with a limited budget, so the method must also be affordable.

Results should also be specific and quantifiable. By knowing *what* the contaminating microbe is and *how much* of it is present, the contamination may be handled appropriately and without unnecessary or excessive costs. False positives could lead to unnecessary product treatment or destruction, while false negatives could allow a contaminating population to persist, grow, and spread. Both situations ultimately lead to financial losses for a brewery, thus it is important for the method of BSM detection to be reliable and accurate.

This paper will discuss the various methods of BSM detection and identification historically and currently used in breweries and the advantages and disadvantages of each. Furthermore, this paper will discuss and present the hypothesis that qPCR is the best currently available method for the routine monitoring, detection, and identification of beer-spoilage microbes for most craft breweries.

## 2. Methods of BSM detection

### 2.1. Culturing

Culturing methods historically have been the main method of microbial detection across all industries, until developments in genomic techniques in the past couple of decades. Most craft breweries with a microbiological testing program currently utilize differential media and culturing techniques to detect growth from surface swabs, brewery water, in wort, and/or in beer at various stages of production. This method is simple and affordable but requires long incubation times and potentially subjective, time-consuming identification processes that come after growth detection (i.e., gram staining, catalase and oxidase assays). Additionally, culturing may have relatively high limits of detection; a single visible colony may be comprised of 10^7^ cells (Berk et al., 2000).

Many commercially prepared versions of differential media for BSMs are available, and most were formulated in the 1960s and ‘70s. Various media formulations have been developed to suppress and encourage growth of specific organisms. Culture media can be incubated either aerobically or anaerobically at specific temperatures for a set amount of time to encourage selective growth conditions.

Lin’s Wild Yeast Media (LWYM) was designed to detect non-*Saccharomyces* wild yeast and suppress growth of pitching yeast and bacteria with fuchin sulphite and crystal violet, though some non-*Saccharomyces* and *Saccharomyces* pitching yeasts may also grow on this media, sometimes giving confusing results (Longley et al., 1980). Shortly after the development of LWYM, Yishan Lin also developed Lin’s Cupric Sulfate Media (LCSM) which he recommended be used in conjunction with LWYM. LCSM supports growth of non-*Saccharomyces* wild yeasts and suppresses growth of *Saccharomyces* pitching yeasts and bacteria with cupric sulfate. LCSM may also detect some *Saccharomyces* yeasts including *S. cerevisiae* variant *diastaticus* (Lin, 1981).

Bacterial detection is often performed using Universal Beer Agar (UBA) (Kozulis and Page, 1968) or Schwarz Differential Agar, also called Lee’s Multi-Differential Agar (SDA/LMDA) (Lee et al., 1975) media containing cycloheximide to suppress pitching yeast growth. Cycloheximide is an antibiotic produced by *Streptomyces griseus* that is a eukaryotic protein synthesis inhibitor and has also been shown to inhibit DNA synthesis in yeast, while having no effect on bacteria (Morris, 1966). Cycloheximide at a concentration of 10 ppm inhibits yeast growth in media (Hill, 2015). It is worth noting that brewery technicians should be aware of the toxic effects of cycloheximide on eukaryotes (including humans) and should take appropriate care in the handling and disposal of this compound.

UBA formulators Kozulis and Page set out to develop a “truly universal microbiological medium to be used in brewing microbiological control,” and stated, “During our investigation in which eventually 17 different media formulations were developed and tested, it was found that tomato juice and beer were the main factors responsible for the excellent characteristics of the new medium.” Tomato juice had previously been shown to stimulate growth of *Lactobacilli.* The beer helped inhibit non-beer microbes while also encouraging growth of BSMs. Though wild yeast may be detected with UBA lacking cycloheximide, so will pitching yeast. Thus, UBA is not useful for cycloheximide-sensitive wild yeasts unless all pitching yeast has been removed or killed by pasteurization. However, UBA containing cycloheximide is useful for bacterial BSM detection (Kozulis and Page, 1968).

In 1975, S.Y. Lee and others from the Adolph Coors Company (Golden, CO) (now part of Molson Coors Brewing Company) utilized similar ingredients as those in UBA (tomato juice and peptonized milk) to develop LMDA. The key difference was the addition of calcium carbonate, bromocresol green, and other ingredients to “promote differences in colony characteristics, such as color, surface appearance, and possible formation of a halo zone around bacterial colonies” (Lee et al., 1975). Formulators outlined the difference in growth appearance of various genera (Lee et al., 1975), however, many analysts may find these appearance differences difficult to interpret and further analysis (e.g., gram stain, microscopic observation) may still be required to determine microbe identity.

MacConkey agar containing cycloheximide is a pH/color-indicating differential media used for the detection of gram-negative bacteria, namely *Enterobacteriaceae* in wort, pitching yeast and/or fermentation vessels. Growth of gram-positive bacteria is inhibited by bile salts and crystal violet, while lactose is the only source of carbon. Neutral red is used as a pH indicator. Fermentation of lactose on media will produce acids that lower the pH, making the colony pink in color. Lactose fermenting (“Lac positive”) species include *Enterobacteriaceae* and *Klebsiella.* Non-lactose fermenting (“Lac negative”) species such as *Pseudomonas* and *Proteus* will not cause a pH change so colonies will remain white (Hill, 2015; Jung and Hoilat, 2021).

Hsu’s *Lactobacillus* and *Pediococcus* (HLP) medium, also containing tomato juice, has been the most used lactic acid bacteria detection media in the brewing industry. Most commonly, semisolid HLP is aseptically inoculated with beer samples in a sterile culture tube with little to no headspace, as to encourage anaerobic growth (Fischborn et al., 2009; Priest and Campbell, 2009; Hill, 2015). However, traditional methods for the use of HLP have proven insufficient for the detection of hard-to-culture and VBNC LAB (Billon et al., 2019). In recent years, deMan, Rogosa, Sharpe (MRS) media (for *Lactobacilli*) with the addition of catalase incubated anaerobically has been shown to allow the resuscitation and growth of some VBNC LAB within 5 days (Deng et al., 2014).

Following visible growth detection on media, identification methods may include a variety of tests which may help identify the microbe to a genus level. These include gram staining, catalase test, oxidase test, and direct microscopic observation of cell morphology (Priest and Campbell, 2009; Hill, 2015). Although simple and affordable, high limits of detection, long incubation times, inability to detect VBNC LAB, and the subjective nature of culturing techniques, relying solely on culturing for BSM detection can lead to decreased quality in a variety of scenarios. Such scenarios include: product sales pending lab results, contaminated products being sold due to false negative results, and/or wasted or lower quality product due to false positive results. Thus, many breweries in recent years have adopted more rapid detection techniques such as genomic analyses in combination with their culturing protocols (Hill, 2015; Pellettieri, 2015).

Additionally, the microcolony method is a culturing method that is more rapid than conventional plating techniques. The microcolony method can be paired with staining with dyes such as carboxyfluorescein diacetate (CFDA) to detect slow growing lactic acid bacteria in as little as 3 days (Asano et al., 2009). CFDA is hydrolized in a reaction with intracellular esterase, producing fluorescence (Bunthof et al., 2001) This method involves passing beer through a membrane filter, then incubating the filter on Advanced Beer-spoiler Detection (ABD) medium. The membrane filter is then transferred onto a filter paper soaked with CFDA staining buffer which is scanned with a bioimaging system such as the μFinder Inspection System (Asahi Breweries, Tokyo, Japan). This bioimaging system uses an epifluorescent microscope to scan for microcolonies on the filter (Asano et al., 2009).

#### 2.1.1. Viable but nonculturable and hard-to-culture microbes

The main beer-spoilage genera of concern are the lactic acid-producing bacteria, *Pediococcus* and *Lactobacillus*, which have been shown to cause up to 90% of beer-spoilage incidents (Sakamoto and Konings, 2003). LAB are generally gram-positive, non-sporulating, facultative anaerobes which are catalase- and oxidase-negative. Heterofermentative LAB metabolize carbohydrates and produce a mixture of lactic acid (sour flavor), carbon dioxide, acetic acid (vinegar flavor), diacetyl (buttery flavor, oily mouthfeel) (Liu et al., 2016a), and/or ethanol. Homofermentative LAB produce mainly lactic acid (Priest and Campbell, 2009). The production of turbidity/ropiness, acidity, excess gas, and other off-flavors also characterize LAB-spoilage incidents (Deng et al., 2014).

Under stressful conditions, *Lactobacilli* cells may alter their metabolic activities to increase energy production and lower the level of stress. One way they do this is by utilizing pyruvate produced by glycolysis in alternate pathways to make ATP (Papadimitriou et al., 2016; Liu et al., 2016a). Other metabolic changes that may occur in response to stress include the utilization of alternative carbon sources, activation of the proteolytic system, and/or the catabolism of free amino acids (Papadimitriou et al., 2016). Changes in metabolic activity alters the viability and growth of stressed LAB. These adaptations are crucial for survival under stressful conditions and vary among different LAB species (Papadimitriou et al., 2016; Liu et al., 2016a, 2017a,b, 2018b). It has been shown by Junyan Liu and others that several species of *Lactobacillus* including *L. brevis* (Liu et al., 2018b), *L. casei* (Liu et al., 2017b)*, L. acetotolerans* (Liu et al., 2016a), *L. lindneri* (Liu et al., 2017a)*, L. harbinensis* (Liu et al., 2018a), and other LAB (Quirós et al., 2009) are capable of entering into a viable-but-nonculturable (VBNC) state. The identification of the VBNC state of bacteria was first introduced in 1982 by Huai-Shu Xu and others, in the water-borne pathogens, *Escherichia coli* and *Vibrio cholerae* (Xu et al., 1982), and an incomplete list published in 2021 reports 101 species spanning 50 genera that have been shown to enter this state of dormancy (Zhang et al., 2021). Entry into this state as a response to stressful environmental conditions is a survival strategy of many bacteria. By entering the VBNC state, bacteria can adapt to changing environments to survive various stresses.

Conditions such as warm or cold temperatures, oxidative stress, and/or nutrient starvation may cause bacteria to enter the VBNC state (Nowakowska and Oliver, 2013). It has been shown that entry into the VBNC state by *Lactobacilli* in beer can be induced by (1) continuous passage in beer, (2) oxidative stress, and/or (3) low-temperature stress (Suzuki et al., 2006; Deng et al., 2015; Liu et al., 2017b, 2018b). Additionally, it has been shown that oxidative stress may be induced by low temperature stress (Chattopadhyay et al., 2011). In *L. acetotolerans,* no genes have been found which encode oxidative stress response, hence the observation of entrance of *L. acetotolerans* into the VBNC state under cold-stress conditions that increase oxidative stress (Deng et al., 2014; Liu et al., 2016b).

In the VBNC state, cells remain “metabolically active” but are resistant to multiple types of stress (Papadimitriou et al., 2016). However, they may completely lose the ability to grow on routine microbiological media, meaning these cells are in the stationary (rather than growth) phase. Some bacteria, that are not able to grow under normal conditions, may be grown slowly under specific conditions and/or in the presence of resuscitating molecules or stimuli (Oliver, 2005; Fakruddin et al., 2013; Deng et al., 2015; Papadimitriou et al., 2016). Such bacteria may also be referred to as “hard-to-culture.” Resuscitating stimuli reverse the conditions that induced entry into the VBNC state and can restore culturability as well as possibly increase metabolic activity for some bacteria (Oliver, 2005), though it has been shown that VBNC lab retain similar metabolic activity to exponentially-growing cells, making them equally capable of beer-spoilage (Liu et al., 2018a).

Catalase has been shown to remove oxidative stress caused by continuous passage in beer and cold stress of *Lactobacilli* to allow resuscitation and restore culturability of LAB previously in the VBNC state (Deng et al., 2014; Liu et al., 2017a,b, 2018a,b). Other non-enzyme peroxide-degrading (thus, oxidative stress-removing) compounds such as sodium pyruvate and α-ketoglutarate have been shown to restore culturability in *Escherichia coli* (Mizunoe et al., 1999)*, Ralstonia solanacearum* (Imazaki and Nakaho, 2009), and other bacteria (Li et al., 2014). These non-enzyme peroxide-degrading compounds may also reduce oxidative stress and restore culturability of VBNC LAB and would be worth experimenting with for this purpose. Other chemical stimuli shown to resuscitate some VBNC bacteria include glutamate, amino acids, Tween 20, vitamins, metal chelating agents, and quorum sensing signal molecules. Active proteins such as resuscitating-promoting factor of gram-positive bacteria and resuscitating-promoting like protein of gram-negative bacteria, have also been shown to resuscitate VBNC bacteria (Zhang et al., 2021). Though cold stress has been shown to induce the VBNC state of LAB and temperature upshift has been shown to resuscitate some bacterial species including *Vibrio vulnificus* (Smith and Oliver, 2006)*, Escherichia coli*, and *Ralstonia solanacearum*, attempts at this method have failed to resuscitate VBNC LAB under cold stress (Deng et al., 2014; Liu et al., 2017a, 2018a).

Interestingly, it has been shown that the VBNC state may be related to antibiotic persistence of some pathogenic bacteria (Fakruddin et al., 2013; Allen et al., 2019). The VBNC state and antibiotic persistence are both states of bacterial dormancy. Dormancy is the state of a viable bacterium which does not grow and has decreased metabolic activity, despite environmental conditions which support growth (Balaban et al., 2019). Due to increasing concerns about antibiotic resistance and failure and the research that has emerged from those concerns, in 2019 Balaban, et al. published “Definitions and guidelines for research on antibiotic persistence.”

Antibiotic persistence is characterized by the observation of a biphasic killing curve, indicating two subpopulations. In antibiotic persistent populations, some cells die quickly after antibiotic treatment, while tolerant cells survive treatment. Tolerance is defined as cells that do not possess a resistance factor such as an efflux pump, yet survive antibiotic treatment above a minimum inhibitory concentration (MIC), and can be regrown upon antibiotic removal. A persister cell is a tolerant cell from an antibiotic persistent population (Ayrapetyan et al., 2018). In one study, stationary-phase cultures contained higher populations of persister cells than growth (logarithmic)-phase cultures, leading researchers to believe that these two states of dormancy, antibiotic persistence and VBNC, are closely related (Fakruddin et al., 2013; Allen et al., 2019).

Because many breweries use differential culturing techniques as the method of detection for BSMs, and because LAB are the most common cause of beer-spoilage and can enter the VBNC state, spoilage from VBNC or hard-to-culture, LAB pose great risk to the industry (Bokulich et al., 2018). Due to the lack of culturability, several recent studies have focused on developing PCR assays for the detection of beer-spoiling LAB.

Furthermore, because culturing methods have long been the standard for microbe detection in many industries, including pathogen detection in the food industry and medical field, the inability to detect microbes in the VBNC state with culture-based techniques can pose much greater dangers than spoiled beer. Scientists have estimated that nearly 99% of all microbial species (potentially 10^12^ total) are yet to be discovered, with only a small percentage of those discovered being culturable (Bodor et al., 2020). With the relative youngness of both genomic methods and knowledge of the VBNC state, it begs the question, how many bacteria have gone unacknowledged and/or undescribed, and for how long, sneaking by in this elusive state of dormancy (Sambo et al., 2018; Bodor et al., 2020)?

Despite the danger posed by VBNC LAB, relatively little is known about the molecular mechanisms these bacteria employ to enter the VBNC state. Future research could be done on the identification of new genomic targets to be used in PCR which are involved in the stress response and entrance into the VBNC state of LAB. By choosing genetic targets that are involved in and expressed during the VBNC state, new assays (such as reverse-transcriptase (RT)-PCR) may be designed and applied for a wide range of VBNC bacteria detection. Additionally, in future work, these targets may be further examined and used to design genus-, species-, and/or strain-specific primers *via* sequence alignments.

### 2.2. Luminescence: ATP swabs and luminometer

ATP (adenosine triphosphate) tests are used commonly in many industrial settings including state health laboratories, hospitality, drug production, veterinary, food and beverage production, and brewing as a rapid method for the detection of ATP on surfaces and in liquids. The presence of ATP indicates the presence of microorganisms but provides no information on the type or viability of organism(s) present (Hygiena, n.d.; Turner et al., 2010; Hill, 2015). These tests are generally used to monitor sanitation and clean-in-place (CIP) processes.

ATP detection systems utilize bioluminescence via the firefly enzyme, luciferase, to detect the presence of organic material from living or dead organisms. The sample surface is swabbed, and the swab is exposed to a lysis buffer to release ATP, as well as luciferin and luciferase. Luciferin and ATP react in the presence of luciferase, forming luciferyl adenylate and phosphate. Luciferyl adenylate then reacts with oxygen to form AMP, carbon dioxide, and oxyluciferin. Oxyluciferin formed in the electronically excited state then releases a photon, emitting a visible yellow-green light. This light emission is monitored and used to measure the quantity of ATP presence in relative light units (RLUs) (Turner et al., 2010; Hill, 2015).

Although these tests are useful for detecting the presence of ATP (from which microbial contamination may be inferred), they may also detect dead microbes incapable of spoiling beer. The presence of organic material on a surface that has not been properly cleaned may yield such a false positive. ATP detection is affordable, rapid, sensitive, accurate, and these systems are useful in practice to check the cleanliness of surfaces, equipment, and vessels used in brewing. However, they are neither specific nor practical for monitoring the microbial stability of liquid product (Hill, 2015).

The MicroStar™-Rapid Microbe Detection System (RMDS) is an ATP-bioluminescence reaction used to detect microbes in beer. Beer is passed through a membrane filter and the filter is placed on agar medium. After incubation the filter is treated with the rapid microbe detection reagent kit which consists of an ATP extraction reagent and a luminous reagent. Immediately after treatment, the filter is placed in a luminous detector and photons emitted are measured. It was reported that as little as a single cell of yeast or 50 cells of bacteria can be detected with this method. Though quick and effective for microbe detection, false positives are reported to be an issue with this method and it does not offer any identification characteristics (Takahashi et al., 2000).

### 2.3. High-throughput genomic methods

#### 2.3.1. High-throughput sequencing of 16S gene

High-throughput sequencing of the 16S ribosomal RNA (rRNA) gene for prokaryotes or 18S rRNA gene for eukaryotes may be used to accurately identify microbial samples and to characterize microbial communities in beer. Ribosomal RNA genes contain slowly evolving regions that generally have low sequence polymorphisms that can be used for genus identification, but also contain fast-evolving regions which can be used as a species identifier (Sambo et al., 2018). Specific regions or the entire length of the 16S rRNA gene are amplified via PCR with degenerate universal primers, then sequenced, commonly with the Next-Generation Sequencing (NGS) platform, Illumina MiSeq (Illumina, Inc., San Diego, CA) (Britton et al., 2015; Sobel et al., 2017; Sambo et al., 2018; Callahan et al., 2019). While the sequencer may be affordable for some breweries ($99,000 new, quote from Illumina November 18, 2021), especially if purchasing preowned (~$25,000) (LabX, n.d.), with running costs at $18/sample (Illumina, 2023) this method is yet to be affordable enough for routine use in most craft breweries. The Illumina MiSeq utilizes a flow cell to perform massively parallel sequencing. The workflow for this technology is comprised of 4 steps: (1) sample preparation, (2) clustering, (3) sequencing, and (4) data analysis (Illumina, 2016).

Once DNA is isolated, (1) reduced cycle amplification is performed and sequencing adaptors are added to the ends of the DNA fragments. Additional oligonucleotides are added to the ends of the DNA fragments including the sequencing binding sites, indices, and regions complimentary to the flow cell oligos. (2) After DNA preparation each fragment is isothermally amplified on the flow cell which is coated in a “lawn” of oligos complimentary to those added to the DNA fragments during sample preparation. Each of fragment strands are simultaneously clonally amplified through bridge amplification (Illumina, 2016). (3) Sequencing of amplified fragments occurs through a proprietary process called “Sequencing-by-Synthesis” (Illumina, Inc., San Diego, CA), in which fluorescently labeled nucleotides bind to single stranded amplification products, emitting detectable fluorescent signals corresponding to each of the four nucleotides. The emission wavelength and signal intensity determine the base added during the sequencing reaction. Millions of sequencing reactions and reads are completed simultaneously for all identical fragments. (4) Then sequences are pooled based on the indices introduced during sample preparation and reads with similar sequences are clustered. Contiguous sequences are then aligned to the reference genome database for identification (Illumina, 2016). Sequences can then be compared to one or more sequence databases such as RefSeq or GenBank to identify the microbial source of the DNA (Britton et al., 2015; Sobel et al., 2017; Callahan et al., 2019).

This method is often useful for species identification but may not be able to resolve closely related species or strains of spoilage microbes from their non-spoilage relatives because of similarity in their 16 s rRNA genes (Callahan et al., 2019). Relative abundances are unreliable, meaning quantification is not possible with this method. Additionally, it is unlikely that many smaller to mid-sized craft breweries can afford performing sequencing of the 16S gene in-house due to high instrument and running costs. Breweries may sometimes send streaked plates to third-party sequencing labs to identify unknown microbes growing on the media. The ability to directly harvest and purify DNA from beer samples would be necessary to utilize this method for the detection of VBNC microbes that cannot be cultured.

Currently this method works best as a non-routine strategy to identify and differentiate dangerous BSMs from potentially benign non-spoilage microbes found growing in high-cost products, especially those that may need a high level of stability due to consumer aging of the product (e.g., barrel aged brands). However, it is possible that costs of this technology may decrease enough in the future that more breweries may be able to utilize 16S rDNA sequencing methods for routine BSM identification (Sobel et al., 2017). Recently, researchers proposed the use of the portable nanopore-based sequencing device, MinION from Oxford Nanopore Technologies (Oxford, UK), calling the machine a “disruptive innovation in sequencing technology (Kurniawan et al., 2021).” The company website boasts that costs for the machine start at a very affordable $1,000 (Oxford Nanopore Technologies, n.d.-a). However, assay and flow cell ($90–$900 per cell) costs are still too high for routine use in craft breweries. If assay costs become lower in the future this may become more practical for routine microbial monitoring in breweries (Oxford Nanopore Technologies, n.d.-a, b).

#### 2.3.2. Polymerase chain reaction

The popularity of PCR for microbial detection and identification in the brewing industry has become more common in recent years, not only in the highly funded macro breweries, but also in smaller craft breweries. PCR is a method that has a wide variety of applications and is used to amplify small segments of DNA over several (~20–30) cycles of a series of temperature fluctuations of the DNA sample and master mix, generally in a machine called a thermal cycler. These temperature fluctuations allow for denaturation of DNA into two single strands (~95°C), followed by annealing (~55°C), and finally extension (~72°C). Oligonucleotide primer pairs (~20 nucleotide long sequence that is complimentary and specific to the target DNA) present in the master mix bind to single stranded DNA during the annealing stage, and the thermostable Taq DNA polymerase performs DNA extension, allowing for exponential amplification of specific target DNA in a sample.

PCR can be used for the targeted analysis of microbial samples to detect the presence of target DNA, delivering results in as little as 1–2 h. Oligonucleotide primers may be designed to target a specific strain (i.e., *S. cerevisiae* v. *diastaticus*, or antibiotic-resistance bacterial strains) or a broader category (i.e., genus) of microbes. For strain-specific detection, primers would be designed to target a unique region of DNA (i.e., antibiotic resistance genes), while for genus detection, primers would target regions of DNA conserved across, yet unique to, the entire genus.

PCR is advantageous due to its low capital and running costs, its high sensitivity, relative simplicity, tunable specificity, accuracy, and ability to deliver rapid results. However, it is important that breweries perform thorough validation of in-house PCR assays before making quality decisions based on PCR results. Because of its sensitivity and the potential for cross-contamination or inhibitors in PCR samples, it is imperative that brewery labs ensure valid PCR results and utilize positive and negative control samples in PCR reactions to avoid inaccurate results.

##### 2.3.2.1. End-point PCR

End-point PCR uses agarose gel electrophoresis to detect amplicons after thermal cycling is performed. Bands of the appropriate size are identified as the target amplicon, indicating the presence of the target microbe in the PCR sample. In temperature gradient gel electrophoresis (TGGE) PCR products encounter increasingly higher temperatures throughout the gel. Similarly, in denaturing gel electrophoresis (DGGE), DNA is exposed to increasingly higher concentrations of chemical denaturant throughout the gel. Differing DNA sequences will denature at different temperatures or concentrations of denaturant (Manzano et al., 2011). These methods have proved effective at differentiating species or strains of yeast and beer-spoilage bacteria when non-strain-specific primers are used in PCR (Manzano et al., 2005, 2011; Garofalo et al., 2015).

##### 2.3.2.2. Real-time, quantitative PCR

Real-time, quantitative PCR (qPCR) can be especially useful in breweries to detect very small microbial populations, as low as 10 cells in a sample. qPCR is affordable for routine use in most craft breweries: the cost of equipment (thermal cycler) may range from just a few thousand to a few dozen thousand dollars, and running costs can be as low as $1–$3 per sample (Billon, 2018). Additionally, qPCR assays are relatively simple and quick to perform, requiring as little as 10 min to 1 h of benchwork and an additional 1–2 h of thermal cycling. qPCR records amplification signal digitally and allows for quantification of the starting DNA in the sample through analyses that are discussed further in later sections. qPCR requires less equipment, is safer, and delivers quicker results than end-point PCR followed by agarose gel detection. Quantification of target DNA in qPCR can be achieved by performing standard curve assays (Bustin et al., 2009; Real-time PCR handbook, 2016). PCR can be performed on DNA extracted from beer samples, or on DNA from colonies growing on agar media (colony PCR). qPCR can be versatile in that primers can be designed to target a broad range of microorganisms, or a specific strain. Sequence alignments may be used to determine highly conserved regions across multiple species as well as regions of uniqueness amongst a set of micro-organisms belonging to a specific genus for example.

Primer design, efficiency, and assay validation are a crucial components of PCR. The Minimum Information for Publication of Quantitative Real-Time PCR Experiments was published in 2009 in response to a lack of standardization and consensus on how to perform, interpret, and report results of qPCR experiments. This useful publication contains guidelines on the minimum information needed for qPCR experiment evaluation as well as a checklist for authors to assess prior to initial manuscript submission to publishers. The authors proposed standard nomenclature and abbreviations as well as conceptual considerations such as accuracy and repeatability, guidelines for sample acquisition/handling/preparation, data analysis, assay validation, and relevant definitions (Bustin et al., 2009).

Assays may be designed with various types of fluorophores, including oligonucleotide probes such as TaqMan® probes and molecular beacons, or with non-specific DNA-intercalating dyes such as SYBR Green. SYBR green is a fluorescent DNA-intercalating dye that binds to all double-stranded DNA during a qPCR reaction. Because of this non-specificity, melt curve analysis should be performed in SYBR Green assays to identify amplification and detection of non-specific targets caused by sample contamination, primer mis-binding, and/or the formation of primer dimers. Amplification of non-specific targets analyzed without melt-curve analysis may lead to false positive results in SYBR Green qPCR assays (Real-time PCR handbook, 2016).

**Figure 1:**
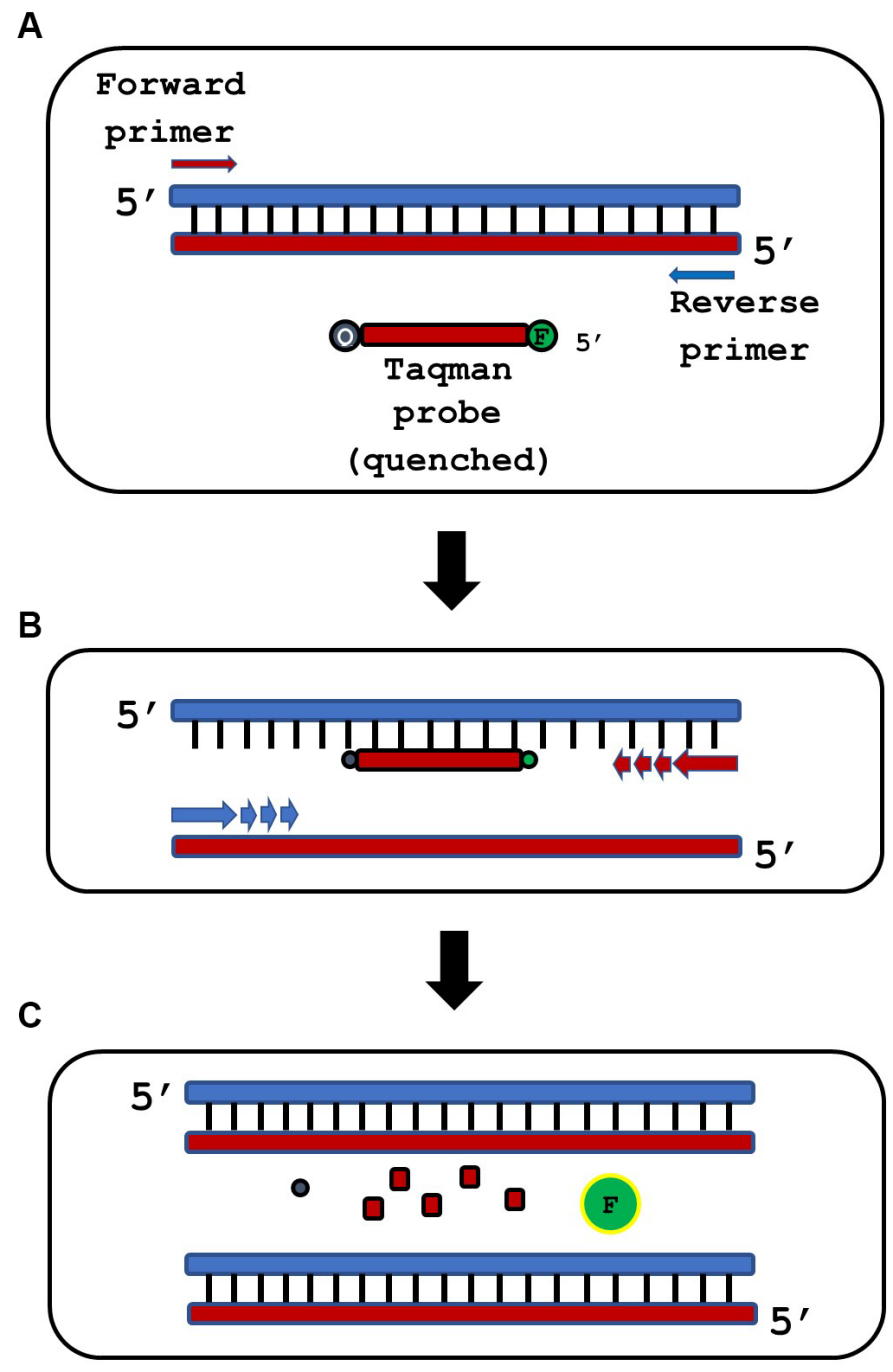
Overview of qPCR using a TaqMan® probe strategy. **(A)** qPCR reactions are assembled containing specific forward and reverse primers and a short, single-stranded Taqman probe that is end-labeled with a 5′ fluorophore (F) and a 3′ quencher (Q). The physical proximity of F and Q in the probe blocks fluorescence of the probe. **(B)** PCR thermocycling is then performed. Denaturation separates double-stranded DNA, annealing allows the Taqman probe to bind its specific complementary DNA target, and extension produces new complementary DNA strands from the specific forward and reverse primers. **(C)** During extension, Taq polymerase uses its 5′ > 3′ exonuclease activity to hydrolyze the annealed Taqman probe. This releases unquenced fluorophore (green F) in stoichiometric ratio to the target template.

An oligonucleotide probe is a short segment of nucleotides that binds to the target amplicon, which contains a fluorophore molecule at the 5′ end and a quencher at the 3′ end (Taqman probes are illustrated below in Figure 1) (Real-time PCR handbook, 2016). Probes are more specific than SYBR Green but tend to be more complicated to design and add significant cost per reaction. Molecular beacons are similar to Taqman probes, but have a unique sequence flanked by inverted repeats, which allow for a stem-loop structure to form and quench fluorescence when complimentary double-stranded DNA is not present (Shaw et al., 2020). Probes and molecular beacons are used for multiplexed reactions, where multiple probes or beacons with different colors of fluorophore may be used to detect multiple targets during the same thermal cycler run (Gilbride, 2014; Shaw et al., 2020).

In lieu of designing primers or oligonucleotide probes, some breweries choose to utilize commercially available qPCR kits. Several companies have developed commercially available qPCR kits specifically designed to detect a variety of common BSMs, either through single or multi-channel reactions. Multi-channel (multiplex) reactions must use oligonucleotide probes. These kits do not allow flexibility for assay modification and/or optimization. Often these kits are only to be used with a thermal cycler which is also sold by the kit manufacturer, potentially making the user limited to only primer/probe options offered by the kit supplier. Although these kits generally do not require advanced training and are simple to use, primer/probe sequences and assay validation are often proprietary, leaving one to question the validity of results. Additionally, the cost of some kits may be up to 20 times as much as the cost of primers and SYBR Green master mix reagents per sample (Billon, 2018). *Thus, SYBR Green qPCR assays may be a more robust and more affordable option* versus *oligonucleotide probes or pre-made kits for breweries of smaller size and on a limited budget.*

###### 2.3.2.2.1. Extraction of PCR-ready DNA

Because (by definition) viable-but-nonculturable bacteria can be difficult or nearly impossible to culture on agar media, colony PCR is not very useful when screening for these bacteria. Thus, it is essential to utilize effective methods of extracting DNA that can be used for qPCR or other nucleic acid amplification methods directly from beer samples. An example of a relatively typical method described by Nakamura et al. (2013), employs the use of lyophilization and magnetic beads.

Additionally, there are many low cost commercially available DNA extraction kits that may work well for harvesting inhibitor-free, PCR-ready DNA from beer spoilers. However, still, many published methods that utilize such extraction kits require first culturing the microbes either as a slurry or on agar medium and then extracting DNA from those cultures. This required pre-enrichment or culturing step can add up to several days to the time it takes to obtain results. Much literature exists on BSM specific PCR methods, some of which utilize what they term “direct DNA extraction,” where DNA is amplified from its source without a significant extraction step. Often this includes simply isolating cells by centrifugation or membrane filtration and wash, then heating cells to cause cell lysis (Juvonen and Haikara, 2009; Shaw et al., 2020). However in most publications found on BSM specific PCR methods samples were either sub-cultured, spiked, or enriched prior to DNA extraction potentially adding days to the time it takes to obtain PCR results (Juvonen and Satokari, 1999; Haakensen et al., 2007; Juvonen et al., 2008; Juvonen and Haikara, 2009; Shaw et al., 2020; Kurniawan et al., 2021).

The Bio-Rad InstaGene™ Matrix (Bio-Rad Laboratories, Inc., Hercules, CA) kit is quick, affordable, easy to perform, and has previously been shown to work well for extracting DNA from beer to be used in PCR (Juvonen and Satokari, 1999; Haakensen et al., 2007; Juvonen et al., 2008; Juvonen and Haikara, 2009). This kit is comprised of 6% w/v Chelex resin which binds PCR inhibitors in beer and products from cell lysis, but does not bind DNA. With this kit and most others, a concentration step is necessary first when harvesting cells from cultures and contaminated beer samples. Concentration may be performed by centrifugation, membrane filtration, or lyophilization. Concentrated samples are mixed with the Chelex matrix in a microcentrifuge tube, heated to cause cell lysis, then centrifuged. DNA is then harvested from the supernatant and is ready for PCR (Bio-Rad US, n.d.).

The Qiagen DNeasy Kit (Qiagen, Hilden, Germany) has many variations for various sample types that have been used in the beer industry for different purposes. Protocols for these kits are relatively simple and quick and start by lysing pelleted cells with lysis buffer and/or beating beads and a vortex adapter. Lysed cells are centrifuged and the DNA-containing supernatant is treated with solutions which utilize patented Inhibitor Removal Technology® (IRT) to remove proteins and PCR inhibitors. DNA is then bound to a silica spin filter which is then washed. Finally DNA is recovered with an elution bufferiagen (Qiagen, 2013–2023a, b). The DNeasy PowerSoil Pro Kit has been used to characterize microbial communities of hops with 16S sequencing (Allen et al., 2019). However, this kit is very costly at $6.66/sample (Qiagen, 2013–2023b). In 2021 Shayevitz and others published a study of the microbiome of barrel aged beers that used the much more affordable DNeasy PowerFood Microbial Kit ($0.37/sample) (Qiagen, 2013–2023a) to harvest DNA from pelleted barrel aged samples also to be used for 16S rRNA sequencing of the present microbes.

One study on the use of the portable nanopore-based sequencing device, MinION from Oxford Nanopore Technologies (Oxford, UK), researchers used the PrepMan™ Ultra reagent (Thermo Fisher Scientific, 2006–2023) to extract DNA from microbial colonies grown on agar media (Kurniawan et al., 2021). “PrepMan™ Ultra reagent is a novel formulation and is a homogeneous solution that does not contain Chelex® or any other type of resin or matrix” (Thermo Fisher Scientific, 2006–2023). It is instructed to be used with culture broths or microbial colonies (Thermo Fisher Scientific, 2006–2023), though it would be interesting to see if it would work for concentrated beer samples.

The Zymo Research DNA Fecal/Soil Microbe MiniPrep Kit (Zymo Research, Irvine, CA) has been used in some studies for extracting beer microbe DNA for PCR (Bokulich et al., 2015; Sobel et al., 2017). This kit is similar to the others, where it starts by collecting sample pellets and then lysing cells via bead beating. It also uses proprietary inhibitor removal technology. Following bead beating, the supernatant of the initial lysis is filtered through a spin filter, and then the filtrate is lysed again with lysis buffer. The resulting liquid is then passed through a spin column that adsorbs DNA. DNA in the column is washed, then eluted with elution buffer, and then passed through a spin filter once more. The filtrate is then ready for PCR or other methods. The Zymo Research DNA Fecal/Soil Microbe MiniPrep Kit protocol is quick and relatively simple like the other kits mentioned but is on the higher end of the cost spectrum ($4.32/sample) (Zymo Research, 2023).

**Figure 2:**
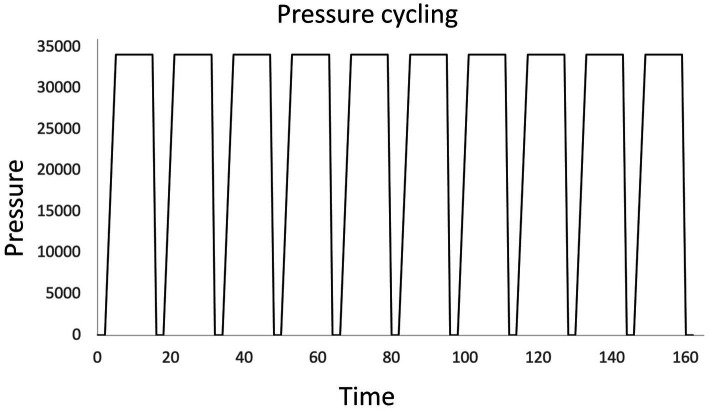
High efficiency DNA extraction from beer samples using a Barocycler. Rapid pressure fluctuation improves the extraction of DNA from samples concentrated by filtration on complex porous membranes, such as mixed cellulose ester membranes (Suzuki, 2020; Shimokawa et al., 2023).

Few publications were found which reported methods for harvesting DNA for PCR directly from beer samples without an enrichment or culturing step (Bokulich et al., 2012, 2015). Some studies directly extracted DNA from centrifuged beer samples for 16S and 18S rRNA sequencing (Sobel et al., 2017; Shayevitz et al., 2021). Pressure cycling technology (PCT) is a cell lysis technique that when used in combination with membrane filtration may increase yield of DNA extraction from filters, though there is little peer-reviewed research on the method. Originally developed as a tool to help extract trace amounts of DNA from forensic samples in complex matrices such as bone or hair, the Barocycler™ (Pressure Biosciences, South Easton, Easton, MA) has also been tested for direct DNA extraction from beer samples. The Barocycler™ subjects samples to rapid pressure cycling, reaching pressures up to 45,000 psi. One cycling treatment method moves from a pressure of 0.1 MPa to 235 MPa, and vice versa, in just seconds as shown in Figure 2 (Suzuki, 2020).

Previous attempts at direct DNA extraction from beer using mixed cellulose ester filters had low recovery rates, as DNA and cells stuck in the membrane filter matrix may be hard to recover. By subjecting mixed-cellulose ester filters containing cells from beer samples to PCT however, higher amounts of DNA were recovered. One study showed that this method was able to extract, detect, and identify only a few cells of BSM *Lactobacillus brevis* in 3000 mL of beer with PCR (Suzuki, 2020). PCT may be combined with an extraction kit to remove PCR inhibitors from the pressure treated samples if necessary. Though the use of an extraction kit may still be required to remove PCR inhibitors, PCT may still be advantageous for improving DNA extraction directly from beer and eliminating the need for a pre-enrichment or culturing step.

Unfortunately the cost of a new Barocycler™ is likely unaffordable for most small or medium sized breweries at $65,000 (price obtained from manufacturer representative as of November 17, 2021). However, preowned models in excellent condition may be available for as little as $3600 (a price even many small breweries could afford) (Ebay search, 2021). Further research on methods for PCR-ready DNA extraction directly from beer samples is needed, and PCT combined with membrane filtration may be a valuable tool with further validation studies. Testing and comparing various DNA extraction methods and kits for harvesting DNA for PCR directly from beer, especially of VBNC BSMs, could be very useful for the brewing industry.

###### 2.3.2.2.2. qPCR primer design and validation and data analysis

There are several considerations to take when designing primers for development of accurate and robust qPCR assays. It is important that researchers take the proper measures in designing and validating qPCR experiments so that results are accurate and reproducible The MIQE Guidelines is a useful publication that contains guidelines on the minimum information needed for qPCR experiment evaluation as well as a checklist for authors to assess prior to initial manuscript submission to publishers. The authors proposed standard nomenclature and abbreviations and included conceptual considerations such as accuracy and repeatability, sample acquisition/handling/preparation, data analysis, assay validation, and several definitions (Bustin et al., 2009). Primer design is a critical aspect of robust, accurate qPCR and may being performed in accordance with the workflow outlined by Bustin and Huggett (Bustin and Huggett, 2017).

*In silico* design involves (1) target identification and (2) the definition of assay properties. Once target gene sequences are identified and collected, NCBI Nucleotide BLAST is performed to check for target uniqueness within the target organism’s own genome as well as against other related species and organisms.

The genomic target can be very specific (e.g., a certain microbial strain) or broad (e.g., an entire genus), and primers may be designed to target a specific gene of the target microbe. If qPCR primers are designed to target certain genes that may be expressed or suppressed under varying conditions and in various physiological states of a microbe, the same primers used for qPCR to detect a BSM may also be used in reverse transcriptase-PCR (RT-PCR) assays to study gene expression. It is possible that these primers can be used to target genes which are upregulated or over-expressed in the VBNC state may be used as markers to identify the induction of the VBNC state of bacteria.

##### 2.3.2.3. Free convective PCR

**Figure 3:**
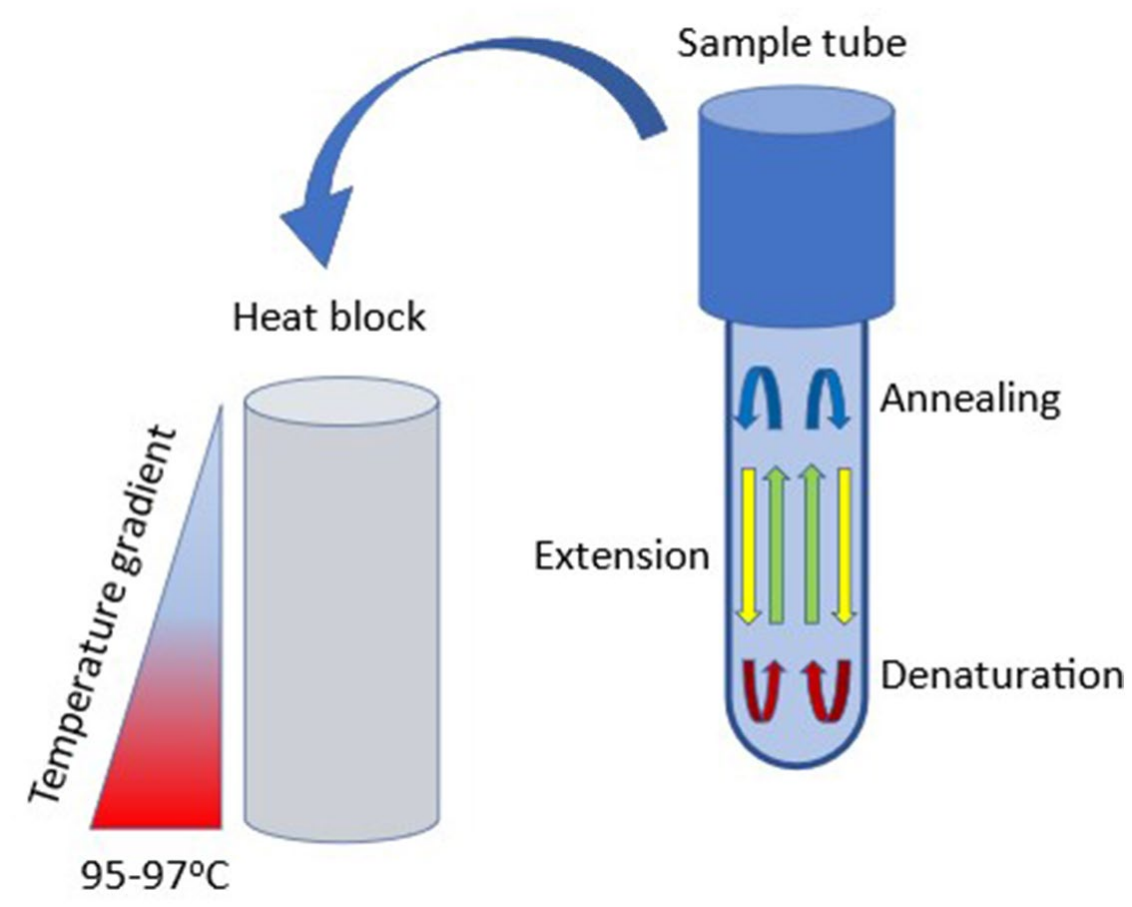
Simplified schematic of free convective PCR. A temperature gradient is applied across a sample tube using a specialized heating block. High temperature is applied at the base of the sample tube and a cooler temperature is applied at the stem, thereby inducing convective (laminar) flow. Under optimal conditions, denaturation occurs at the base of the tube, annealing toward the top, and primer extension occurs along the shaft.

First described in 2001, free convective PCR (cPCR) is a novel continuous-flow-based microfluidic PCR technique that requires no thermal cycler or external pumps making it more affordable than traditional PCR. Additionally, due to its high amplification efficiency it is able to deliver results in less time than traditional PCR (Rajendran et al., 2019; Miao et al., 2020). Because of these qualities, cPCR has been investigated as a Point of Care Test (POCT) for rapid detection and identification of certain microorganisms in the medical field, such as antibiotic-resistant bacteria (Rajendran et al., 2019). In cPCR, a steady-state vertical temperature gradient is created by heating the capillary tube (often the bottom, to 95°C) to induce spontaneous thermal convection in the tube. The liquid in the reaction vessel is stratified into spatially separate and stable temperature zones that allow for DNA melting, annealing, and extension, as shown in Figure 3 (Miao et al., 2020).

Many variables can be tuned in cPCR depending on the specific assay requirements or desired traits. cPCR may be performed under either laminar or chaotic advection modes, and it has been shown that the chaotic advection mode can improve PCR amplification efficiency. Furthermore, there are several different reactor types that can be used in cPCR, such as capillary tubes, disks, and closed loops (Figure 3). Capillary tube-based systems are currently the simplest to operate and set up. Another important aspect of cPCR is the method of thermal control used, either contact or no-contact heating. Several heating modules have been developed for capillary tube systems (Miao et al., 2020).

Traditionally the largest hurdle in using cPCR in POCTs has been the post-PCR amplification detection method, such as agarose gel electrophoresis. In recent years, quantitative cPCR assays have been developed for POCTs that utilize a smartphone camera with the appropriate LED filters to detect and measure fluorescence (Rajendran et al., 2019; Miao et al., 2020). One study combined cPCR with a lateral flow assay that used quantum dots as fluorescent labels and a smartphone camera for amplification detection to detect antibiotic resistant bacteria (Rajendran et al., 2019). Additionally, though it will not be discussed in any detail, it is worth noting that carbon quantum dots are another novel tool that have recently been employed in microbial detection, as fluorescent tags and more, including antibacterial applications, that may soon become more commonly used with further research (Anand et al., 2019). Recently, Khodakov et al. published a study in which researchers developed a portable, battery powered cPCR device and an assay using this device which was able to detect as low as 10 copies of DNA in only 30 min, making cPCR with this device even more attractive as a POCT (Khodakov et al., 2021). Due to the low cost of equipment and rapidness of results without compromising the sensitivity or sensitivity of traditional PCR methods, with appropriate assay development and validation cPCR may be useful in detection and identification of BSMs.

#### 2.3.3. Isothermal amplification methods

With the application of appropriate DNA extraction methods, such as those discussed above, each of the following isothermal amplification methods could be used to detect and identify beer spoilage microbes, including those in the VBNC state.

##### 2.3.3.1. Loop-mediated isothermal amplification

Loop-mediated isothermal amplification (LAMP) is a method that utilizes four to six primers that recognize six to eight distinct regions of DNA and a strand-displacing DNA polymerase which initiates synthesis of target DNA. This is a highly specific amplification reaction which happens isothermally, so no thermal cycling is required, and because the polymerase is strand-displacing, no initial DNA denaturation step is required. Two primers form loop structures. Extension of the loops and annealing of the additional primers allows for more amplification of target DNA. DNA products of this process are >20 kb repeats of the target sequence (80–250 bp) connected with single-stranded loop regions (New England Biolabs, 2023).

Modes of target DNA detection include: real-time fluorescence with intercalating agents (e.g., SYBR green) or probes, lateral flow, or agarose gel. Real-time fluorescence is used for quantitative measurements (New England Biolabs, 2023). Additionally, after the reaction takes place and DNA is amplified, white precipitates comprised of magnesium pyrophosphate are found in the reaction mixture. Thus, real-time monitoring of the LAMP reaction can be performed by real-time monitoring of turbidity of the reaction mixture, where the amount of turbidity directly corresponds to the amount of target DNA amplified (Tsuchiya et al., 2007). All that is required for LAMP instrumentation is consistent heating to the desired assay temperature with equipment and detection equipment (New England Biolabs, 2023).

In 2007 Tsuchiya et al. used LAMP PCR for the detection of BSMs. Primers were designed for four beer-spoilage bacteria (*Lactobacillus brevis*, *Lactobacillus lindneri*, *Pediococcus damnosous*, and *Pectinatus*) and used the method to identify isolated colonies grown on agar medium. They performed the multiplex reaction at 65°C, and determined the limit of detection of this assay to be six copies of target DNA at the start of the reaction (Tsuchiya et al., 2007). Additionally, LAMP has been used to detect and identify *Lactobacillus brevis, L. lindneri, L. backii,* and *Paracollinoides*. Target species or strains were distinguished from other non-target lactic acid bacteria in 40–60 min, with a detection limit of 100 CFU/mL (Murakami et al., 2009). LAMP could be useful in a brewery setting as a rapid and affordable method of BSM detection and identification.

##### 2.3.3.2. Polymerase spiral reaction

Polymerase spiral reaction (PSR) is a cost-effective nucleic acid amplification method that uses one pair of primers, one enzyme, Betaine, and a strand-displacing DNA polymerase in an isothermal reaction that occurs at 61–65°C in about 1 h. Betaine destabilizes the DNA double helix which helps facilitate primer binding and DNA amplification. Liu et al. (2015) reported that the method can detect as low as six CFU per reaction. This study used PSR to detect *E. coli* containing the *bla*_NDM-1_ gene. The *bla*_NDM-1_ gene is 813 bp long and was chosen as the target sequence because it is a super antibiotic resistant gene. Positive amplification results were detected with agarose gel and also by SYBR Green dye fluorescence in an qPCR instrument. The reaction can also be monitored with a real-time turbidimeter. The forward and reverse rimer sequences are reverse to each other at their 5′ ends, while at their 3′ ends they are complementary to their respective target nucleic acid sequences. The product of PSR is a spiral structure. Primer design for PSR is simple with primer design software. Reagents can be mixed and stored at −20°C like Master Mix for polymerase chain reaction (Liu et al., 2015). Due to these characteristics, PSR would likely work well for detection of BSMs, though further research specifically for BSM detection is warranted.

##### 2.3.3.3. Cross-priming amplification

Cross-priming amplification (CPA), like several other isothermal amplification methods, uses a strand displacing DNA polymerase, eliminating the need for an initial DNA denaturation step. The reaction occurs at 63°C in less than an hour and is more cost-effective than polymerase chain reaction. The cross primers have 5′ ends that are not complementary to the template strand, and a displacement primer binds to the target upstream of the crossing primer. Strand displacement occurs due to annealing of the cross primers. In CPA amplification is highly specific and sensitive with a limit of detection as low as 4 bacterial cells (Xu et al., 2012). No research was found using CPA to detect BSMs, but being affordable, quick, specific, and sensitive, CPA could work well for detection of BSMs in the brewing industry.

##### 2.3.3.4. Rolling circle amplification

Like many of the isothermal amplification methods listed above, rolling circle amplification (RCA) uses two primers and a strand displacing DNA polymerase. RCA is performed at room temperature or 30°C and is highly sensitive and specific, with a femtmolar limit of detection, able to detect one copy of target DNA in 100,000 copies non-target DNA with a one to two base discrimination, and can be completed within an hourAmplification is based on a circular DNA template and the product of RCA is a long single stranded DNA molecule comprised of tandem repeats of the target sequence. Ligation-RCA (L-RCA) is a variation of RCA based on a padlock probe that is designed to hybridize with the target sequence. Probes that hybridize with the target sequence then undergo ligation to form circular DNA templates which can then be amplified through RCA (Goo and Kim, 2016). By coupling RCA with padlock probes these reactions can be multiplexed (Soares et al., 2021). Reagents for RCA are very affordable (Molecular Cloning Laboratories, 1998–2023). Yin et al. (2018) used RCA to detect the viable but nonculturable beer spoilage *Lactobacillus acetotolerans*, targeting the gene, *horA*, which encodes for hop-resistance of beer-spoilage lactic acid bacteria. Their method was rapid, sensitive, and specific, and lower cost than PCR, showing that RCA can be an effective tool for BSM detection (Yin et al., 2018).

##### 2.3.3.5. Recombinase polymerase amplification

Recombinase polymerase amplification (RPA) is an isothermal amplification reaction that takes place at 37–42°C with the use of two primers, a recombinase protein, and a strand displacing DNA polymerase. Primer design is the same as it is for polymerase chain reaction. RPA is highly sensitive and selective, requires minimal sample preparation, and can multiplex reactions. RPA starts when the recombinase protein binds to primers in the presence of ATP and a crowding agent, forming a recombinase-primer complex. This complex then intercalates the double-stranded DNA and the primer binds as the complementary site. The recombinase-primer complex dissociates, and the strand-displacing DNA polymerase binds to the 3′ end of the primer, extending the DNA strand in the presence of dNTPs. This process is cyclically repeated for exponential amplification of target DNA. RPA reagents are more costly than PCR reagents, but equipment costs for RPA are lower than that of PCR. While no research was found on the use of RPA for detection of BSMs, this method would be effective, although it is not as cost effective as some of the other isothermal amplification techniques discussed (Lobato and O’Sullivan, 2018).

#### 2.3.4. Fluorescence *in situ* hybridization and flow cytometry

Fluorescence *in situ* hybridization (FISH) and flow cytometry (FCM) are fluorescent probe-based techniques that may be paired with one another to detect, identify, and quantify microbes in beer samples and other microbial samples. Previously used in wine and more recently in brewing research, FISH-FCM is a targeted analysis technique which uses fluorescently labeled nucleic acid probes corresponding to specific DNA sequences, most commonly rDNA, of microbes of interest. Similar to multiplex PCR assays that utilize TaqMan® probes or molecular beacons, FISH DNA probes can be labeled with different fluorophores to detect multiple organisms simultaneously in the same sample. Though still relatively complicated and expensive, FISH probes are easier and less expensive to design than FCM probes or antibodies, while FCM allows for automatic separation and quantification of cells, making the pairing of the two methods more efficient than either method on its own in brewery applications. Additionally, because FISH-FCM allows cells to be visualized *in situ* there is no need for a DNA extraction step (Bokulich et al., 2018).

The morphology of cells in various states of dormancy such as VBNC or persister cells is often different than that of active cells (Oliver, 2005; Bodor et al., 2020; Power et al., 2021). A study of *Lactobacillus acetotolerans* demonstrated by scanning electron microscopy that cells slowly morphed from short rods to coccoids and became smaller in size upon entrance into the VBNC state (Deng et al., 2015). FISH-FCM is able to characterize cell morphology, and thus can be used to identify dormant cells in a population (Power et al., 2021). However, due to the high cost of a flow cytometer, this method is not within the budget of most craft breweries, though it may be useful in research settings for profiling of microbial communities in beer (Bokulich et al., 2018). Because FISH-FCM is a targeted analysis, the user can only detect microbes they are intentionally screening for (via DNA probes).

#### 2.3.5. Mass spectrometry-based proteomics profiling

Matrix-assisted laser desorption/ionization time-of-flight mass spectrometry (MALDI-ToF-MS) is a type of spectrometry capable of identifying large biological molecules and microbes. Proteomics, the study of proteomes and their functions, has been used with MALDI-ToF-MS to characterize peptides and proteins from microbial sources (Cañas et al., 2006). Mass profiles of ribosomal proteins in a sample, some of which are unique to specific microbes (Suzuki, 2020), or whole microbial cells (Holland et al., 1996) are detected and measured by the spectrometer.

Acquired spectra are compared to mass fingerprints to identify the source of the proteins or cells (Gao et al., 2021). Comparison fingerprints are provided by reference databases or can be constructed by the user with authentic control samples (Suzuki, 2020). Because replicate experiments may not yield exact spectra matches with reference samples due to variation in results caused by differing equipment, operators, geographical location, experimental set-ups, etc., fingerprint comparison can be difficult (Holland et al., 1996; Singhal et al., 2015). Additionally, new microbial isolates for which no authentic positive control samples are available may only be identified if mass fingerprints of the specific isolate are available in reference databases. Due to the necessity of well-stocked reference databases, MALDI-ToF-MS currently struggles to distinguish some closely related microbes to a species or strain level (Singhal et al., 2015). Reference methods such as 16S rRNA sequencing may be used to identify new isolates in conjunction with MALDI-ToF-MS to help build reference databases.

Another drawback to MALDI-ToF-MS is that it is difficult to use for identification of microbes in polymicrobial samples because peaks from multiple species may merge into a single peak in the mass spectrum. Results of polymicrobial samples often are only able to identify the predominant organism. Few researchers have reported identification with MALDI-ToF-MS of polymicrobial samples. More research is needed to increase accuracy and precision of analysis of polymicrobial samples with MALDI-ToF-MS (Florio et al., 2019).

Quantification of detected compounds can be performed but requires standard samples for calibration. This can be an issue for unknown samples where the analyst does not yet know what molecules are present. Isotopically labeled versions of molecules of interest can be used to create a standard calibration curve. However, it has been shown that linearity may not exist at more than roughly 5-fold concentration difference, so preliminary measurements must be made to estimate the concentration range of the compounds present before creating the calibration line used for exact quantification (Szájli et al., 2008).

Advantages of this method are that it is rapid and high-throughput, versatile, inexpensive running costs (less than $1 per sample), and it is an effective way to identify cells in which are in the viable-but-nonculturable state (Suzuki, 2020). However, due to the disadvantages mentioned above, and that most breweries cannot afford the initial capital investment costs for MALDI-ToF-MS equipment, MALDI-ToF is currently not feasible for most breweries to utilize for accurate, routine, in-house sample analyses. Breweries may opt to send samples to third party labs for MALDI-ToF-MS analysis non-routinely, though some sample analyses may require isolated colonies grown on agar media (Charles River Laboratories, 2021; Creative Biogene, 2023).

## 3. Discussion

Viable-but-nonculturable lactic acid bacteria pose the greatest risk for product spoilage in the brewing industry. It’s been shown that LAB are the cause of the majority of beer-spoilage cases (Sakamoto and Konings, 2003), most commonly *Lactobacillus brevis* (Menz et al., 2010). Several LAB species have been shown to have the capability of entering the VBNC state (Quirós et al., 2009; Liu et al., 2016a, 2017a, 2018a,b). The traditional methods of detection for these bacteria involve the culturing of aseptically collected samples from throughout the brewing process and often deliver false negative results for hard-to-culture and VBNC bacteria. In addition, selective media is not guaranteed to detect all BSMs, even if culturable on some types of media (Table 1).

**Table 1:** Summary of described methods of BSM detection and identification and their utility in VBNC BSM detection and identification and in most breweries.

Method	Can detect and identify VBNC BSMs in beer	Feasible for use in small-medium sized breweries
Culturing	No	Yes
Luminescence	No	Yes
16S rRNA sequencing	Yes	No
End-point PCR	Yes	Yes
qPCR	Yes	Yes
cPCR	Yes	Yes
LAMP	Yes	Yes
PSR	Yes	Yes
CPA	Yes	Yes
RCA	Yes	Yes
RPA	Yes	Yes
FISH-FCM	Yes	No
MS-based proteomics	Yes	No

*Saccharomyces cerevisiae* variant *diastaticus* is culturable on some types of media but may go undetected on other types and can quickly cause problems when gone undetected. When given time and the ability to reproduce, this strain can wreak havoc on a brewery by potentially growing and spreading rapidly through yeast cultures. Breweries must be capable of detecting and identifying BSMs throughout the entire process of producing, packaging, and selling beer. The risk of contamination spreading and ultimately product and financial losses to the brewery decreases the sooner a contamination is detected.

On the other hand, false positive detection could also lead to unnecessary waste. Brewery labs must be able to accurately detect the presence of BSMs and to quantify the amount present as rapidly as possible, often on a limited budget (Pellettieri, 2015). Real-time, quantitative PCR is a useful tool in the brewing industry due to its ability to deliver rapid results. SYBR Green assays offer simplicity and low costs. However, thorough validation of primers and PCR assays must be done to ensure accurate and reliable results, which manufactured beer spoiler qPCR kits may not be able to offer (Bustin et al., 2009).

Additionally, because enrichment methods increase detection time and may be futile for cultivating VBNC bacteria, valid DNA extraction methods must be used to obtain genomic DNA from beer samples that can be used for PCR. qPCR as a method for detecting BSMs is rapid, specific, accurate, quantifiable, sensitive, and affordable. Additionally, qPCR is relatively simple to perform. With thorough and careful target identification, primer and assay design and validation, validation of DNA extraction methods from beer and new primer sets, qPCR can be employed as a useful tool for BSM detection and identification in most breweries.

The craft brewing industry could greatly benefit from further research and publications on DNA extraction methods for obtaining PCR-ready BSM DNA directly from beer and the design of qPCR primers targeting the ever-evolving number and variety of BSMs. DNA extraction methods that require pre-enrichment increase time required for BSM detection and methods that require culturing are not useful for detecting VBNC microbes. To limit detection time, future BSM detection research should focus on direct DNA extraction methods that isolate cells by centrifugation or membrane filtration paired with a DNA extraction kit such as the affordable, DNeasy PowerFood Microbial kit.

When qPCR is paired with validated DNA extraction methods it can be used to detect VBNC bacteria which go undetected by traditional culturing methods commonly routinely used in quality control and assurance in the food and beverage industry, as well as in water treatment, pharmaceutical and medical fields06 Thus, it may be useful to design qPCR primers targeting genes involved in the VBNC state. These primers can then also be used in RT-PCR assays to study the factors contributing to and involved in the VBNC and/or antibiotic persistence state(s) of dormancy in bacteria. Targeted bacteria could range from beneficial to contaminants to pathogens. As one may imagine, SYBR Green qPCR can be a valuable tool used for microbe detection and identification in many industries besides brewing, such as medical, pharmaceutical, water treatment, biological, virology, epidemiology, forensics, food and beverage, agricultural, and other fermentation processes. cPCR is also a promising method that does not require a thermal cycler, decreasing equipment costs for breweries, though further research is needed in the area.

Paired with valid DNA extraction techniques, isothermal amplification methods are also promising tools for detection and identification of BSMs. These methods do not require a thermal cycler like PCR, decreasing equipment costs, as reactions take place at a single temperature and can be performed in a heating block. LAMP requires 4–6 primers (Tsuchiya et al., 2007) and CPA requires multiple primers including at least cross primer making CPA assay design more complex than some other methods (Xu et al., 2012). Reactions can be multiplexed with RCA, though this requires designing padlock probes (Soares et al., 2021). PSR is a method that requires only two primers and a turbidimeter can be used to monitor amplification (Liu et al., 2015). Though further research using PSR to detect BSMs is needed, this method may work well as a simple and affordable detection method in brewery settings. RPA also requires only two primers that are designed in the same way that PCR primers are designed, and RPA reactions can be multiplexed. However, RPA is more costly than the other isothermal amplification methods discussed (Lobato and O’Sullivan, 2018). Similarly to PSR, no research was found that uses RPA to detect and identify BSMs, though research in this area could be beneficial to the brewing industry.

Currently sequencing of the 16S rRNA gene, FISH-FCM, and MALDI-ToF-MS are too expensive for the majority of craft breweries to employ, so research should not be focused in this area. Overall, future research in BSM detection ad identification should focus on utilizing the well-established SYBR Green qPCR method as well as various isothermal amplification methods that are lower cost than PCR.

## Author contributions

RO performed the literature review and research and wrote the manuscript. MH reviewed and edited the manuscript. All authors contributed to the article and approved the submitted version.

## Funding

This research was funded by the Held Laboratory, Ohio University, Department of Chemistry and Biochemistry and Jackie O’s Brewery, Athens, OH, USA.

## Conflict of interest

The authors declare that the research was conducted in the absence of any commercial or financial relationships that could be construed as a potential conflict of interest.

## Publisher’s note

All claims expressed in this article are solely those of the authors and do not necessarily represent those of their affiliated organizations, or those of the publisher, the editors and the reviewers. Any product that may be evaluated in this article, or claim that may be made by its manufacturer, is not guaranteed or endorsed by the publisher.
